# Substitution at Aspartic Acid 1128 in the SARS Coronavirus Spike Glycoprotein Mediates Escape from a S2 Domain-Targeting Neutralizing Monoclonal Antibody

**DOI:** 10.1371/journal.pone.0102415

**Published:** 2014-07-14

**Authors:** Oi-Wing Ng, Choong-Tat Keng, Cynthia Sau-Wai Leung, J. S. Malik Peiris, Leo Lit Man Poon, Yee-Joo Tan

**Affiliations:** 1 Department of Microbiology, Yong Loo Lin School of Medicine, National University Health System (NUHS), National University of Singapore, Singapore; 2 Institute of Molecular and Cell Biology, A*STAR (Agency for Science, Technology and Research), Singapore; 3 Centre of Influenza Research, School of Public Health, The University of Hong Kong, Hong Kong, China; German Primate Center, Germany

## Abstract

The Severe Acute Respiratory Syndrome Coronavirus (SARS-CoV) is the etiological agent for the infectious disease, SARS, which first emerged 10 years ago. SARS-CoV is a zoonotic virus that has crossed the species barriers to infect humans. Bats, which harbour a diverse pool of SARS-like CoVs (SL-CoVs), are believed to be the natural reservoir. The SARS-CoV surface Spike (S) protein is a major antigenic determinant in eliciting neutralizing antibody production during SARS-CoV infection. In our previous work, we showed that a panel of murine monoclonal antibodies (mAbs) that target the S2 subunit of the S protein are capable of neutralizing SARS-CoV infection *in vitro* (Lip KM *et al*, J Virol. 2006 Jan; 80(2): 941–50). In this study, we report our findings on the characterization of one of these mAbs, known as 1A9, which binds to the S protein at a novel epitope within the S2 subunit at amino acids 1111–1130. MAb 1A9 is a broadly neutralizing mAb that prevents viral entry mediated by the S proteins of human and civet SARS-CoVs as well as bat SL-CoVs. By generating mutant SARS-CoV that escapes the neutralization by mAb 1A9, the residue D1128 in S was found to be crucial for its interaction with mAb 1A9. S protein containing the substitution of D1128 with alanine (D1128A) exhibited a significant decrease in binding capability to mAb 1A9 compared to wild-type S protein. By using a pseudotyped viral entry assay, it was shown that the D1128A substitution in the escape virus allows it to overcome the viral entry blockage by mAb 1A9. In addition, the D1128A mutation was found to exert no effects on the S protein cell surface expression and incorporation into virion particles, suggesting that the escape virus retains the same viral entry property as the wild-type virus.

## Introduction

The Severe Acute Respiratory Syndrome (SARS) first emerged as an infectious disease ten years ago, manifesting itself as a severe form of pneumonia. Its etiological agent was identified as a then novel coronavirus known as the SARS coronavirus (SARS-CoV) [1], [2]. Within a short span of time from December 2002 to July 2003, the newly emerged virus spread quickly to infect more than 8000 people across 25 countries with an overall fatality rate of approximately 10% [3]. SARS-CoV is a zoonotic virus that has crossed the species barrier to infect humans. Small animals such as palm civets (*Paguma larvata*) and raccoon dogs (*Nyctereutes procynonoides*) sold in live-animal wet markets in Guangdong Province of Southern China are believed to be the zoonotic source of the virus transmitted to humans [4]. In 2005, the complete sequences of SARS-like coronaviruses (SL-CoVs) of genetic homology of 87–92% to SARS-CoV were identified from horseshoe bats of the genus *Rhinolophus* in China [5], [6]. However, these SL-CoVs display significant differences in sequences at the receptor-binding domain (RBD) compared to SARS-CoV and are unable to use the SARS-CoV receptor, the human angiotensin-converting enzyme 2 (ACE2), for cellular entry [7], rendering them unlikely to be the immediate progenitor of SARS-CoV. More recently, a bat SL-CoV capable of using the human ACE2 receptor for cellular entry was characterized and isolated from Chinese horseshoe bats, providing strong evidence that bats are the natural reservoirs of SARS-CoV [8].

The SARS-CoV is classified as a virus from the genus betacoronavirus (lineage B), family *Coronaviridae* and order *Nidovirales*. It is an enveloped, positive-sense, single-stranded RNA virus of genome size of approximately 29.7 kb, encoding for 16 non-structural proteins, 8 accessory proteins and 4 structural proteins (namely the spike [S], envelope [E], membrane [M] and nucleocapsid [N] proteins) [9], [10], [11]. Every surface spike of the SARS-CoV is composed of a trimer of S protein of 1255 amino acids in length. The S protein is a type 1 glycoprotein and a class 1 fusion protein responsible for viral attachment and entry into host cells, and is therefore the principal determinant of host range [12]. It consists of 2 functional subunits: the N-terminal S1 subunit (amino acids 15–679) and the C-terminal S2 subunit (amino acids 680–1255). The S1 subunit contains the RBD [13], [14] that recognizes the SARS-CoV receptor, ACE2 [15], allowing the attachment of the virus to its host cell. The S2 subunit contains the putative fusion peptide and two heptad repeats, HR1 and HR2, important in SARS-CoV fusion with target host cells [16], [17]. Upon the association of the RBD with the ACE2 receptor, a conformational change of S2 is triggered, resulting in the insertion of the fusion peptide into the target cell membrane [18], followed by the interaction of the HR1 and HR2 domains in an anti-parallel manner to form a stable six-helical bundle fusion core [17], [19]. This allows the viral membrane and target cell membrane to come into close proximity, facilitating membrane fusion and viral entry [20], [21].

**Figure 1:**
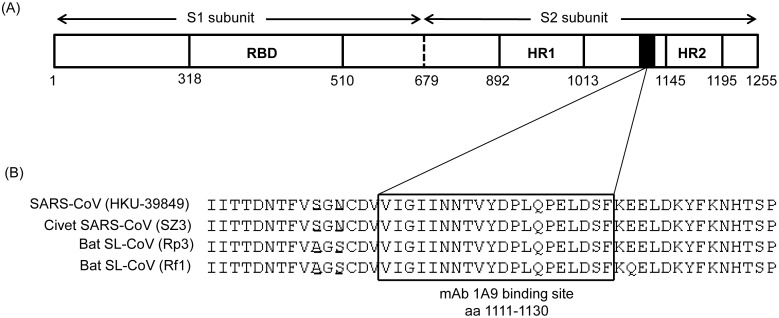
Binding site of mAb 1A9 within the SARS-CoV S protein. (A) Schematic diagram of the different motifs in the SARS-CoV S protein. RBD, receptor binding domain; HR1, heptad repeats 1 domain; HR2, heptad repeats 2 domain. Black box represents the domain in S that is required for the interaction with mAb 1A9. (B) The S region corresponding to the binding site of mAb 1A9 (boxed) to human SARS-CoV HKU39849 strain is aligned with that of civet SARS-CoV SZ3 strain, bat SL-CoV Rp3 and Rf1 strains. Two residues upstream of this binding site are not conserved and they have been underlined.

Besides its roles in viral entry, the S protein is also a major antigenic determinant in eliciting humoral immune responses in infected humans [22] and is therefore an important target in the development of vaccines and therapeutic intervention against SARS [23], [24]. Numerous human and murine monoclonal antibodies (mAbs) binding to the S1 or S2 regions of the S protein have been identified and were shown to confer neutralizing activities *in vitro* and protection *in vivo* against SARS-CoV infection [25], [26], [27], [28]. The S1 subunit of the S protein, especially the RBD, is highly variable among coronaviruses, resulting in a wide range of tissue tropism, while the S2 subunit is a well-conserved domain, indicating the highly conserved nature of the fusion process [29]. As a result, anti-S2 mAbs have broadly neutralizing characteristics against a wider range of SARS-CoV variants, including human and zoonotic SARS-CoV strains, through the recognition of highly-conserved epitopes [28], . In our previous study, it has been shown that a panel of murine mAbs targeting the HR2 domain and the region upstream of HR2 of the S protein are capable of neutralizing SARS-CoV infection *in vitro* [31]. In this study, the cross-neutralization ability of one of these mAbs, termed as 1A9, was investigated by studying its ability to prevent viral entry mediated by the S protein of SARS-CoV strain from civet as well as SL-CoV strains from bats. MAb 1A9 binds to the loop region between the HR1 and HR2 domains and this region is highly conserved but has no known function (Figure 1). In addition, escape mutants against mAb 1A9 were generated to identify critical residues important for mAb 1A9 binding to S protein. We found that mAb 1A9 has broad cross-neutralizing activity and identified a single amino acid (aspartic acid) at position 1128 in S to be crucial for the interaction with mAb 1A9. By using a pseudotyped viral entry assay, it was shown that the substitution of this residue with alanine (D1128A) mediates escape from mAb 1A9 neutralization. In addition, the D1128A mutation exerts no effects on the expression of S on the cell surface and its incorporation into virion particles, suggesting that the escape virus retains the same viral entry property as the wild-type virus.

## Materials and Methods

### Ascites production

Ascites were produced by injecting hybridoma cells into the peritoneal cavities of pristine-primed BALB/c mice. The protocol was approved by the Institutional Animal Care and Use Committee (IACUC) of the Biological Resource Centre, A*Star, Singapore (Protocol Number: 110694). All the procedures were carried out in strict accordance with the recommendations of the National Advisory Committee for Laboratory Animal Research (NACLAR) guidelines in Singapore. All efforts were made to minimize suffering and euthanasia was performed using carbon dioxide.

### Cells and virus

Vero E6 (American Type Culture Collection) and 293 FT cells (Invitrogen) were grown in Dulbecco’s modified Eagle’s medium (Invitrogen) supplemented with 10% fetal bovine serum (Hyclone), nonessential amino acids (Gibco) and penicillin (10,000 units/ml)-streptomycin (10 mg/ml) solution (Sigma Aldrich). CHO cell line stably expressing the human ACE2, known as CHO-ACE2, was established previously [31], and cultured in the same medium. All cell lines were maintained at 37°C with 5% CO_2_. The human SARS-CoV strain HKU39849 was used in this study.

### Purification of monoclonal antibodies

Antibodies were purified from the ascites by using affinity chromatography. Briefly, a 1 ml HiTrap Protein G HP beads column (GE Healthcare) was pre-washed using ∼20 ml of 20 mM sodium phosphate buffer, pH 7.0 at a constant flow-rate of 1 ml/min using a peristaltic pump. 5 ml of ascites fluids were mixed with equal volume of 40 mM sodium phosphate buffer and passed through a 0.45 µm filter. The filtered ascites fluids were passed through the column at the same flow-rate of 1 ml/min. Extensive washing was performed using the 20 mM sodium phosphate buffer. Elution buffer (0.1 M glycine-HCl, pH 2.7) was then passed through the column at a flow-rate of 1 ml/min and the flow-through was collected at 0.5 ml fractions in 1.5 ml microtubes containing 20 µl of neutralization buffer (1 M Tris-HCl, pH 9.0). The concentration of the purified monoclonal antibodies in each tube was determined using the Coomassie Plus protein assay reagent (Thermo Scientific).

### Generation of escape mutants

The generation of escape mutants was performed in a biosafety level 3 (BSL-3) laboratory. Using 100 TCID_50_ of SARS-CoV (strain HKU39849) for infection of Vero E6 cells in the presence of different concentrations of mAb 1A9, the concentration of mAb 1A9 that reduced the virus titers by about 4 logarithms (log) was determined to be 0.25 mg/ml and used for the generation of virus escape mutants. Serial dilutions of SARS-CoV ranging from 10^−1^ to 10^−8^ were incubated in the presence of 0.25 mg/ml of mAb 1A9 for 1 h at 37°C and 5% CO_2_. The virus-mAb mixtures were then incubated with Vero E6 cells in a 96-well plate for 1 hour at 37°C and 5% CO_2_, after which the virus-mAb mixtures were removed and the cells were washed twice with medium. The cells were further incubated for 2 days in the presence of mAb 1A9 at concentration 0.25 mg/ml. The supernatant from the wells containing cells that exhibited cytopathic effect (CPE) at the highest dilution of SARS-CoV was harvested. The percentage CPE was determined by visual counting of floating cells and attached cells. Wells with more than 80% floating cells are considered to have CPE. This supernatant was again incubated in the presence of 0.25 mg/ml of mAb 1A9 for 1 h at 37°C before the virus-mAb mixture was used to infect Vero E6 cells in the presence of mAb 1A9 at concentration 0.25 mg/ml. This was performed 3 times. The final virus sample was added to Vero E6 cells in a 6-well plate and incubated for 1 hour at 37°C and 5% CO_2_ before the wells were overlaid with agarose containing 0.25 mg/ml mAb 1A9 and incubated for 3–5 days at 37°C and 5% CO_2_. Five plaques were picked using a pasteur pipette, freeze-thawed once and further amplified in Vero E6 cells. Neutralization tests were then performed on all the virus clones to confirm that they could escape neutralization by mAb 1A9.

### TOPO cloning and sequencing

Viral RNA of the 5 escape virus clones was isolated using the QIAamp viral RNA mini kit (Qiagen) and converted into cDNA by standard reverse transcription (SuperScript II Reverse Transcriptase, Invitrogen). The cDNA was then amplified by PCR using specific primers targeting the S gene to generate a long fragment (amino acids 1 to 1003) and a short fragment (amino acids 969 to 1255). These gene fragments were cloned into pCR2.1-TOPO vector (Invitrogen) and five colonies were sequenced.

### Construction of plasmids for expression in mammalian cells

The S gene of the human SARS-CoV HKU39849 strain was obtained from viral RNA after reverse transcription and PCR and cloned into the pXJ3′ expression vector using the BamHI and XhoI restriction sites (pXJ3′-S). Chimeric S genes of civet SARS-CoV SZ3 strain, bat SL-CoV Rp3 and Rf1 strains were designed by replacing their receptor-binding domain (RBD) with that of the human SARS-CoV HKU39849 strain. For SZ3, the arginine (R) residue at position 344 was substituted with lysine (K); serine (S) residue at position 360 was substituted with phenylalanine (F); lysine at position 479 was substituted with asparagines and serine at position 487 was substituted with threonine (T). For Rp3 and Rf1, the RBD domain of amino acids 322–496 was replaced with the RBD domain (amino acids 318–510) of the human HKU39849. They were then chemically synthesized (GenScript USA Inc., Piscataway, NJ) and cloned into the same expression vector by using the same restriction sites.

To generate plasmids for the expression of mutant S, specific primers were designed for two-round PCR site-directed mutagenesis of wild-type S gene using the Expand High Fidelity PCR System (Roche). The PCR products were then cloned into the pXJ3′ expression vector using BamHI and XhoI restriction sites to form pXJ3′-S-N1056K, pXJ3′-S-D1128A, pXJ3′-S-D1128A/N1056K, pXJ3′-S-D1128E and pXJ3′-S-D1128N plasmids.

### Western Blot analysis and immunoprecipitation (IP)

Expression plasmids expressing wild-type and mutant human SARS-CoV S, chimeric civet SARS-CoV SZ3 S, bat SL-CoV Rp3 and Rf1 S genes were transiently transfected into 293 FT cells using Lipofectamine 2000 reagent (Invitrogen) according to manufacturer’s protocol. The cells were harvested at 24 hours post-transfection by scrapping and cells were spun down by centrifugation and washed with cold PBS twice. Cell were then resuspended in lysis buffer (50 mM Tris [pH 8.0], 150 mM NaCl, 0.5% NP40, 0.5% deoxycholic acid, 0.005% SDS and 1 mM phenylmethylsulfonyl fluoride) and subjected to freeze-thaw five times followed by spinning down at 13,000 rpm to remove cell debris. Cell lysate protein concentrations were quantitated using the commassie plus protein assay reagent (Thermo Scientific) for use in Western blot analysis and immunoprecipitation (IP). In Western blot analysis, proteins were separated on a 7.5% polyacrylamide gel by sodium dodecyl sulfate polyacrylamide gel electrophoresis (SDS-PAGE) and transferred onto nitrocellulose membranes. The membranes were blocked in 5% skimmed milk in tris-buffered saline (TBS) with 0.05% Tween 20 (TBST) and incubated with primary antibodies, mAbs 1A9 and 7G12 [31] overnight at 4°C. The membranes were then washed in TBST before incubation with goat anti-mouse horseradish peroxidase (HRP)-conjugated antibody (Pierce) as the secondary antibody at room temperature for 1 hour. The membranes were washed in TBST again followed by the addition of enhanced chemiluminescence substrate (Pierce) for film development.

In IP, mAbs 1A9 and 7G12 were used to pull down wild-type and mutant S proteins in the cell lysates for 1 hour at 4°C, followed by the addition of protein A beads (Roche) and incubation at 4°C overnight. The beads were then washed in lysis buffer three times and subjected to Western blot analysis for the detection of S proteins using the rabbit anti-SΔ1 antibody (binds to amino acids 48–358 of the S1 subunit) [32] as primary antibody and goat anti-rabbit HRP-conjugated antibody (Pierce) as secondary antibody.

### Expression and purification of S fragments in bacteria

A fragment of S(1030-1188 aa) was expressed as a GST (glutathione-transferase) fusion protein using the pGEX6p1 vector (GE healthcare). This fragment is located in the S2 subunit at amino acids 1030-1188 and contains the mAb 1A9 binding site. The wild-type and mutant (N1056K and D1128A) S fragments were expressed in *Escherichia coli* BL21-DE3. Cultures were grown in Terrific Broth and on reaching an optical density at 600 nm (OD600 nm) of 0.8, cells were cooled to 16°C and induced with isopropyl *β*-D-thiogalactopyranoside (IPTG) at a final concentration of 0.5 mM. After an incubation period of 24 hours, cells were harvested. Bacterial pellets were resuspended in lysis buffer containing 20% sarkosyl and subjected to sonication. The lysate was cleared by centrifugation and incubated with gluthathione (GSH) sepharose beads (GE Heathcare) overnight at 4°C. After several washes, the GST-tagged S fragments were eluted from the beads in 10 mM reduced gluthathione solution (Sigma Aldrich). Purified S fragments were then subjected to SDS-PAGE on a 12% gel and stained using coomassie blue to visualize the purity of proteins.

### Enzyme-linked immunosorbent assay (ELISA)

Purified wild-type and mutant (N1056K and D1128A) GST-S(1030–1188 aa) proteins were coated onto 96-well ELISA plates (Nunc) overnight at 4°C at 100 ng/well. The wells were blocked in 5% skimmed milk in phosphate-buffered saline (PBS) with 0.1% Tween 20 (PBST) for 1 hour at room temperature, and primary antibodies (mAb 1A9 and mouse anti-GST antibody [Santa Cruz]) were added as primary antibodies at 4-fold dilutions and incubated at 37°C for 2–3 hours. The wells were then washed in PBST followed by the addition of goat anti-mouse HRP-conjugated antibody (Pierce) as secondary antibody and incubated at 37°C for 1 hour. Tetramethylbenzidine substrate (Pierce) was added and reaction was stopped using 0.2 M sulphuric acid. Optical density at 450 nm (OD450 nm) was obtained using an absorbance reader (Tecan Infinite M200). Statistical difference in binding of mAb 1A9 to wild-type S and mutant S was analysed using unpaired t-test. Significance was indicated by *p*-value of <0.01.

### Generation of pseudotyped particles expressing S on the surface (S-pp)

The ability of SARS-CoV containing mutant S to infect cells and the resulting effect in mAb neutralization in SARS-CoV entry were studied using a pseudotyped virus system. Based on this pseudotyped virus system, replication incompetent lentiviral particles expressing S proteins on the surface and containing the firefly luciferase reporter gene were used in replacement of live SARS-CoVs. Viral entry into permissive cell lines will be reflected in the luciferase activity of the infected cells. To generate S-pseudotyped particle (S-pp), lentiviral vector pNL43-R^-^E^-^Luc and plasmids expressing S genes were co-transfected in 293 FT cells using Lipofectamine 2000 reagent (Invitrogen) according to manufacturer’s protocol. 48 hours post-transfection, the viral supernatant was collected and spun down in a centrifuge to remove cell debris. P24 ELISA (QuickTiter Lentivirus Titer kit, Cells Biolabs) was used according to manufacturer’s protocol to quantify viral titres.

### *In vitro* S-pp neutralization assay

All S-pp neutralization assays were carried out in 24-well plates. CHO-ACE2 cells were grown in 500 ul of growing media per well for 24 hours before each experiment. In S-pp neutralization assays, 16 ng of S-pp (as quantified using P24 ELISA) were pre-incubated with mAb 1A9 or mAb 1G10 at 0, 25, 50, 100, 150 and 200 µg/ml for 1 hour at room temperature. The mAb-virus mixtures or virus alone were used to infect CHO-ACE2 cells and incubated at 37°C. A non-neutralizing anti-S1 antibody that binds to the RBD of S, mAb 7G12 [31], was used as a control antibody at 200 µg/ml. At 48 hours post-infection, cells were harvested using the luciferase assay system (Promega) and luciferase expressions of the cells were determined according to manufacturer’s protocol. Percentages of viral entry were then calculated based on the luciferase readings obtained. All experiments were carried out in triplicates. Statistical difference in viral entry between wild-type and mutant S-pp was done using unpaired t-test. Significance was indicated by *p*-value of <0.01.

### ELISA for quantifying the amount of S protein in S-pp

S-pps were coated onto 96-well ELISA plates (Nunc) at 16 ng/well (as quantitated P24 ELISA) overnight at 4°C. The wells were blocked in 5% skimmed milk in PBS with 0.1% Tween 20 (PBST), and primary antibodies (mAb 7G12 [31] and mouse anti-P24 antibody) were added as primary antibodies at 4-fold dilutions and incubated at 37°C for 2 hours. The wells were then washed in PBST followed by the addition of goat anti-mouse HRP-conjugated antibody (Pierce) as secondary antibody and incubated at 37°C for 1 hour. Tetramethylbenzidine substrate (Pierce) was added and reaction was stopped using 0.2 M sulphuric acid. OD450 nm was obtained using an absorbance reader (Tecan Infinite M200). Difference in S protein level in wild-type and mutant D1128A S-pp was evaluated using unpaired t-test.

### Fluorescence-activated Cell Sorting (FACS) analysis for surface expression of S protein

293 FT cells were seeded in 6-cm dishes 24 hours prior to transfection. The cells were transfected with pXJ3′ empty vector, pXJ3′-S and pXJ3′-S-D1128A plasmids using Lipofectamine 2000 reagent (Invitrogen) according to manufacturer’s protocol and harvested at 72 hours post-transfection. The cells were first detached using the cell dissociation solution (Sigma), washed twice in 1x PBS and incubated with purified mouse mAb 7G12 [31] (that binds to the S1 subunit) in 1x PBS containing 1% bovine serum albumin (BSA) for 3 hours at 4°C on a nutator. The cells were washed 3 times using 1x PBS containing 1% BSA and then incubated with fluorescein isothiocyanate (FITC)-conjugated goat anti-mouse IgG (Santa Cruz) secondary antibody for 1 hour at 4°C on the nutator. Cells were washed again 3 times and immediately used for FACS analysis using the CyAn flow cytometer (Beckman Coulter). All FACS data was analysed using the FlowJo software application.

## Results

### *In vitro* neutralization of civet and bat S-pps by mAb 1A9

As described in our previous publication, we have a panel of neutralizing mAbs largely grouped into Type I, II, III and IV based on their binding sites on the S protein. By membrane fusion experiment, we found that mAb 1A9 belonging to Type II was the most effective in cell-cell membrane blocking and bound to residues 1111-1130 which are immediately upstream of the HR2 domain (Figure 1A) [31]. As the contribution of the mAb 1A9 binding site to the structure and function of S has not been defined, we chose mAb 1A9 for further investigation in this study in order to gain a better understanding of the neutralizing mechanism of mAb 1A9.

**Figure 2:**
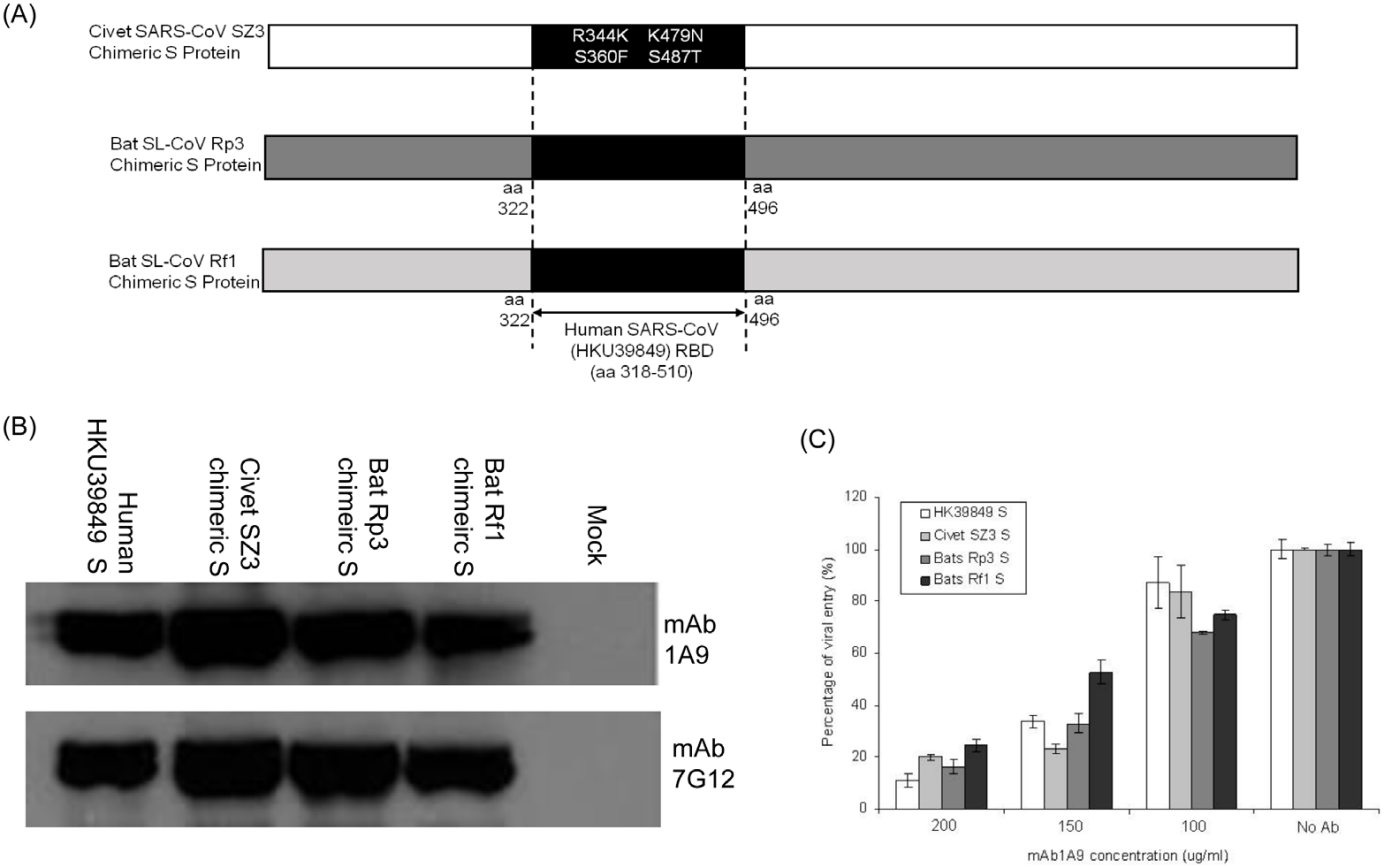
Binding and neutralization of human, civet SARS-CoVs and bat SL-CoVs to mAb 1A9. (A) Schematic representation of chimeric S protein of civet SARS-CoV SZ3, bat SL-CoVs Rp3 and Rf1. The civet SARS-CoV SZ3 chimeric S contains four mutations (R344K, S360F, K479N and S485T) at its RBD that is different from the RBD of human SARS-CoV HKU39849. The bat SL-CoVs Rp3 and Rf1 chimeric S proteins had their entire RBD (amino acids 322–496) replaced by the RBD of human SARS-CoV HKU39849 (amino acids 318–518). (B) 293 FT cells were transfected with no plasmid (mock) or with plasmids expressing S of human SARS-CoV HKU39849, RBD-modified chimeric S of civet SARS-CoV SZ3, bat SL-CoV Rp3 and Rf1 respectively. Western Blot analysis was performed on the cell lysates using mAb 1A9 to determine if it can bind to different S proteins. MAb 7G12, which binds to the S1 of the S protein, was used as a control antibody to detect protein expression. (C) S-pps containing S of human HKU39849, civet SZ3, bat Rp3 or Rf1, were pre-incubated with different concentrations of mAb 1A9 at 100, 150 and 200 µg/ml for 1 hour before infecting CHO-ACE2 cells. Cells were harvested 48 hours post-infection and luciferase activities were measured. Viral entry, as indicated by the luciferase activity measured in relative light units (RLU), was expressed as a percentage of the reading obtained in the absence of antibody, which was set at 100%. Data shown represents that obtained from 3 independent experiments. Bars represent SD of the experiment carried out in triplicates.

Sequence alignment shows that residues 1111-1130 is a highly conserved region within the S2 subunit of human, civet SARS-CoV and bat SL-CoV strains (Figure 1B). It has been demonstrated by Ren *et al*. [7] that the bat SL-CoV Rp3 uses a different unknown receptor for entry and thus, could not infect cells expressing human ACE2 receptor. In order to evaluate the viral entry properties of the civet SARS-CoV SZ3, bat SL-CoV Rp3 and Rf1 strains, the receptor-binding domain (RBD) of S in the civet SARS-CoV SZ3 strain and bat SL-CoV Rp3 and Rf1 strains were replaced with the RBD of S in the human SARS-CoV to create chimeric S proteins (Figure 2A) to allow viral attachment step through the binding of the RBD of S to human ACE2 receptor. By Western blot analysis, mAb 1A9 was found to be able to bind to the S2 subunits of the civet SARS-CoV SZ3 and bat SL-CoV Rp3 and Rf1 strains to similar extent as the homologous human SARS-CoV HKU39849 (Figure 2B). An antibody targeting the S1 domain of the human SARS-CoV S protein, mAb 7G12 [31], was used to determine the relative expressions of the chimeric S proteins (Figure 2B).

Next, to determine if mAb 1A9 exhibits cross-neutralizing activity, S-pseudotyped virus particles, or S-pps, carrying the human SARS-CoV S or the various RBD-modified chimeric S of civet SARS-CoV SZ3 strain and bat SL-CoV Rp3 and Rf1 strains were generated and used to infect CHO-ACE2 cells in the absence or presence of different concentrations (100, 150 and 200 µg/ml) of mAb 1A9. In this pseudotyped virus system, replication incompetent lentiviral particles that express S proteins on the surface were used in replacement of live SARS-CoVs and SL-CoVs. This system has been successfully employed in the study of highly pathogenic viruses including the influenza virus [33] and SARS-CoV [34], [35], [36]. Neutralizing antibody titers measured using pseudotyped SARS-CoV correlated well with the use of replication competent SARS-CoV [37], as such, this system has been widely used in the evaluation of SARS-CoV neutralizing antibodies [30], [38], [39], [40], [41]. In this study, the S-pps expressing human SARS-CoV S or RBD-modified chimeric S of civet SARS-CoV SZ3 strain and bat SL-CoV Rp3 and Rf1 strains were able to infect and enter CHO-ACE2 cells at similar extent (Figure S1A in File S1). The results obtained from the mAb 1A9 neutralization assay (Figure 2C, Figure S2 in File S1) showed that mAb 1A9, apart from being able to neutralize S-pp expressing human SARS-CoV HKU39849 S, was able to cross-neutralize the S-pp expressing chimeric S of civet SARS-CoV SZ3 and bat SL-CoV Rp3 and Rf1. This indicates that mAb 1A9 has broad neutralizing capability.

### Generation of mAb 1A9 escape mutants and identification of S mutations in mAb 1A9 escape mutants

**Figure 3:**
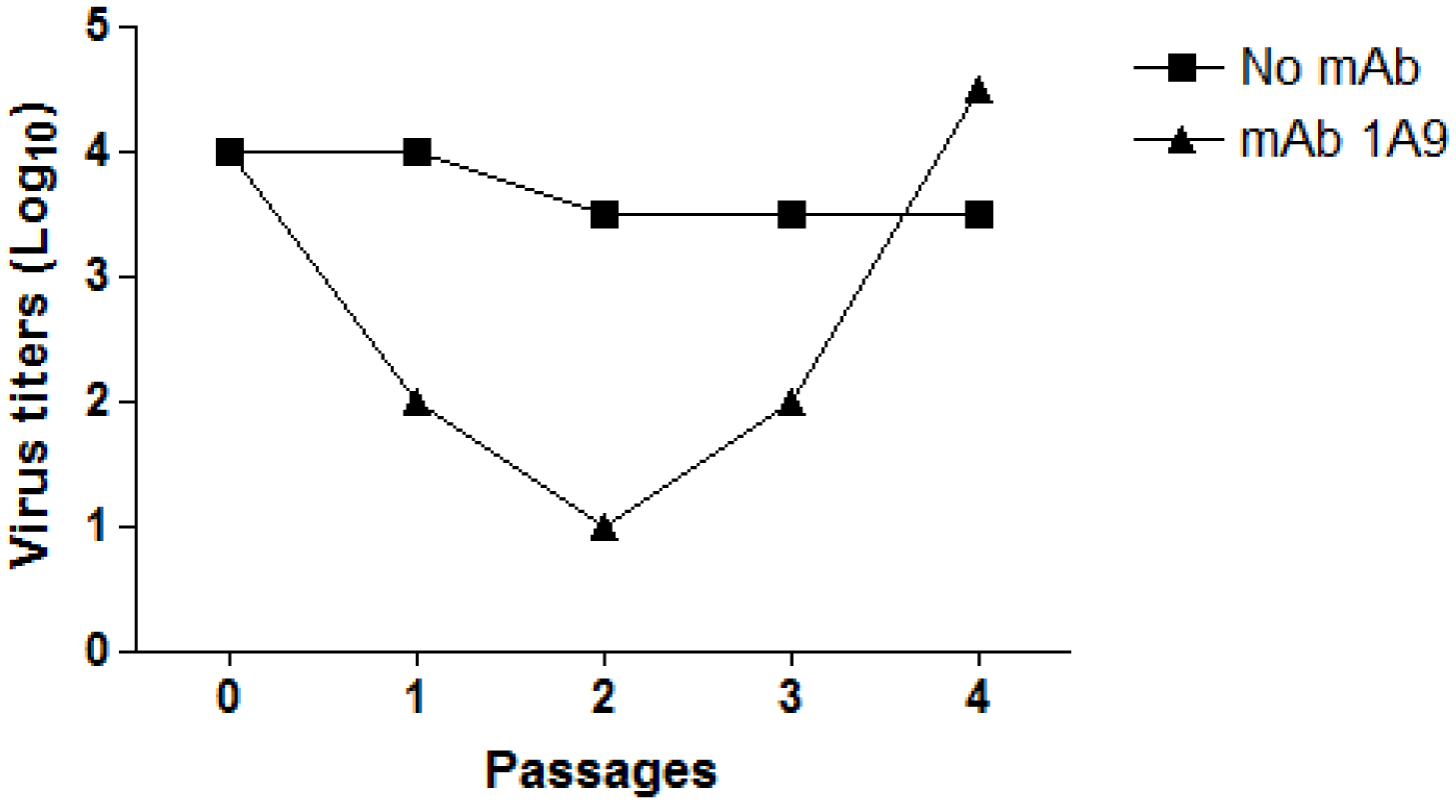
Generation of escape SARS-CoV mutant virus in the presence of mAb 1A9. Human SARS-CoV HKU39849 strain was cultured in Vero E6 cells in the absence or presence of mAb 1A9 (0.25 mg/ml). Supernatant from the wells containing cells that exhibited cytopathic effect (CPE) at the highest dilution of SARS-CoV was harvested as passage 1. The virus was then passaged 3 times in the presence of mAb 1A9 (0.25 mg/ml) and the virus supernatant harvested at each passage was titrated on Vero E6 cells.

To identify critical residue(s) required for mAb 1A9 interaction with S, mAb 1A9 escape mutants were generated. SARS-CoV HKU39849 strain was cultured in Vero E6 cells at a sub-optimal level of mAb 1A9 (0.25 mg/ml). Supernatant from the wells containing cells that exhibited cytopathic effect (CPE) at the highest dilution of SARS-CoV was harvested as passage 1 and passaged 3 times in the presence of mAb 1A9 (0.25 mg/ml), after which the virus titres gradually increased (Figure 3) and a plaque assay was done to isolate 5 individual SARS-CoV escape mutant clones.

**Figure 4:**
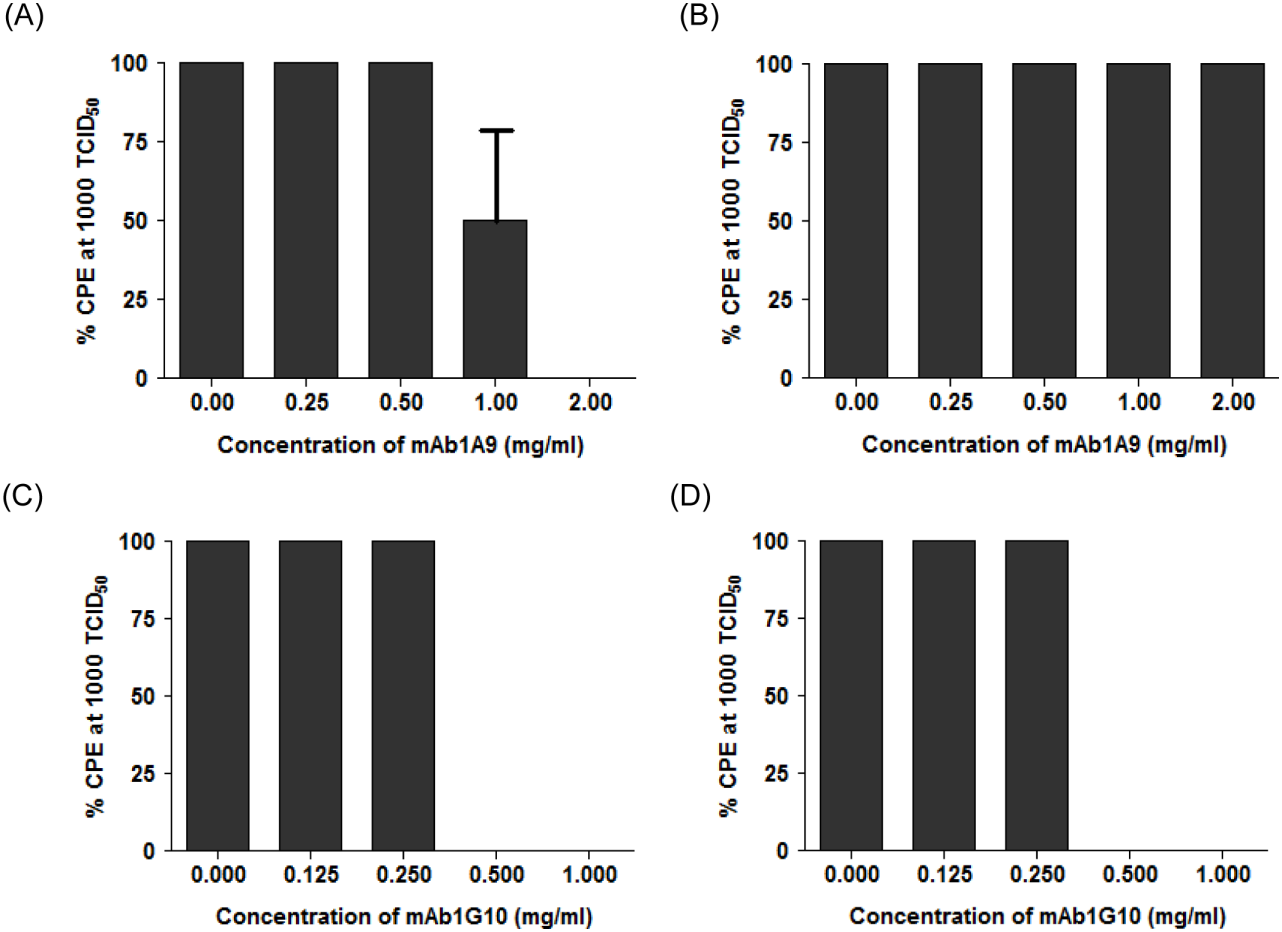
Neutralization of wild-type and 1A9 escape SARS-CoV virus by mAb 1A9 and mAb 1G10. Wild-type human SARS-CoV HKU39849 strain (A and C) and escape mutant SARS-CoV virus generated in the presence mAb 1A9 (B and D) were used at 1000 TCID_50_ to infect Vero E6 cells in the presence of two mAbs, namely mAb 1A9 (A and B) and mAb 1G10 (C and D). MAb 1A9 was used at concentrations 0, 0.25, 0.5, 1.0 and 2.0 mg/ml while mAb 1G10 was used at concentrations 0, 0.125, 0.25, 0.5 and 1.0 mg/ml. Percentage cytopathic effects (% CPE) of the infected cells was observed. MAb 1G10, a SARS-CoV-neutralizing, anti-S2 mAb that binds to S2 at residues 1151–1192, was used as the control mAb.

The wild-type and escape mutant viruses were used at 1000 TCID_50_ and subjected to neutralization assays by mAb 1A9 and mAb 1G10 in Vero E6 cells to determine cytopathic effects (CPE). All 5 escape mutant clones were resistant to neutralization by mAb 1A9 and the result of one representative clone is shown in Figure 4. The percentage of CPE in Vero E6 cells infected with wild-type virus decreased significantly at mAb 1A9 concentrations >1 mg/ml (Figure 4A). In contrast, 100% CPE was still observed in escape mutant virus-infected cells even in the presence of 2 mg/ml of mAb 1A9 (Figure 4B), indicating that the mutant virus is resistant to neutralization by mAb 1A9. However, both the wild-type and mutant virus were equally sensitive to neutralization by another mAb 1G10 (Figure 4C and D), which binds to an epitope (residues 1151–1192) in S2 different from that of mAb 1A9 [31]. After confirming the escape ability of the escape mutant clones, the viral RNA was extracted and the S gene was sequenced. Two escape mutations, N1056K and D1128A, were identified in the mAb 1A9 escape mutants. 2 out of 5 clones had D1128A mutation and 3 out of 5 clones had N1056K mutation. None of the clones had both mutations.

### Difference in mAb 1A9 binding to wild-type and mutant S proteins

**Figure 5:**
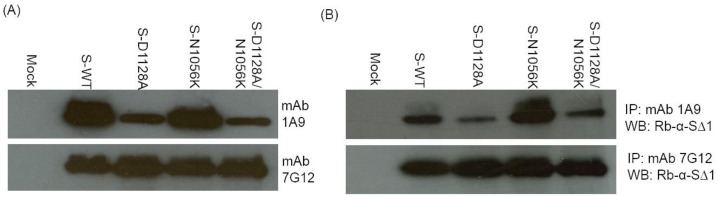
Binding of mAb 1A9 to wild-type, mutant D1128A, mutant N1056K and mutant D1128A/N1056K S proteins by Western Blot and immunoprecipitation. 293(mock) or with plasmids expressing full length wild-type S (S-WT) or full length mutant S (S-D1128A, S-N1056K and S-D1128A/N1056K). (A) Western Blot analysis was performed on the cell lysates using mAb 1A9 to determine if it can bind to the different S proteins. MAb 7G12, which binds to the S1 of the S protein, was used as a control antibody to detect protein expression. (B) Cell lysates containing S-WT, S-D1128A, S-N1056K or S-D1128A/N1056K were subjected to immunoprecipitation (IP) using mAb 1A9 or 7G12 and protein A beads. S proteins immunoprecipitated by mAbs 1A9 and 7G12 were detected using rabbit anti-SΔ1 antibody in Western blot analysis (WB). The rabbit anti-SΔ1 antibody binds to amino acids 48–358 of the S1 subunit.

Wild-type S, substitution S mutants, namely D1128A, N1056K, and that containing both D1128A and N1056K, were then expressed in 293 FT cells and Western Blot analysis was performed to determine the effects of these mutations on the binding of the S protein to mAb 1A9. As shown in Figure 5A, S protein containing mutation D1128A (S-D1128A) showed a reduced binding to mAb 1A9 compared to the wild-type S protein (S-WT), while S protein with the N1056K mutation (S-N1056K) did not show a reduction in mAb 1A9 binding compared to S-WT. While Western Blot reveals the binding of mAb 1A9 to denatured epitope on S, immunoprecipitation (IP) was performed to compare the mAb 1A9 binding to native forms of S. Consistent with the results obtained in Western Blot analysis, S-D1128A exhibited a decrease in mAb 1A9 binding compared to S-WT while S-N1056K did not (Figure 5B). Similar results were also observed in ELISA (Figure S3 in File S1) using wild-type and mutant D1128A and N1056K GST-S(1030-1188) fragments that were expressed from bacteria cells (Figure S4 in File S1). These results suggest that residue 1128 is important in the binding of mAb 1A9 to the S protein. In addition, S protein containing both mutations was evaluated for synergistic effects in the reduction of mAb 1A9 binding by Western Blot and immunoprecipitation. As shown in Figure 5A and B, no significant synergistic effect was observed.

### Resistance of S-pp expressing mutant D1128A S protein to neutralization by mAb 1A9

**Figure 6:**
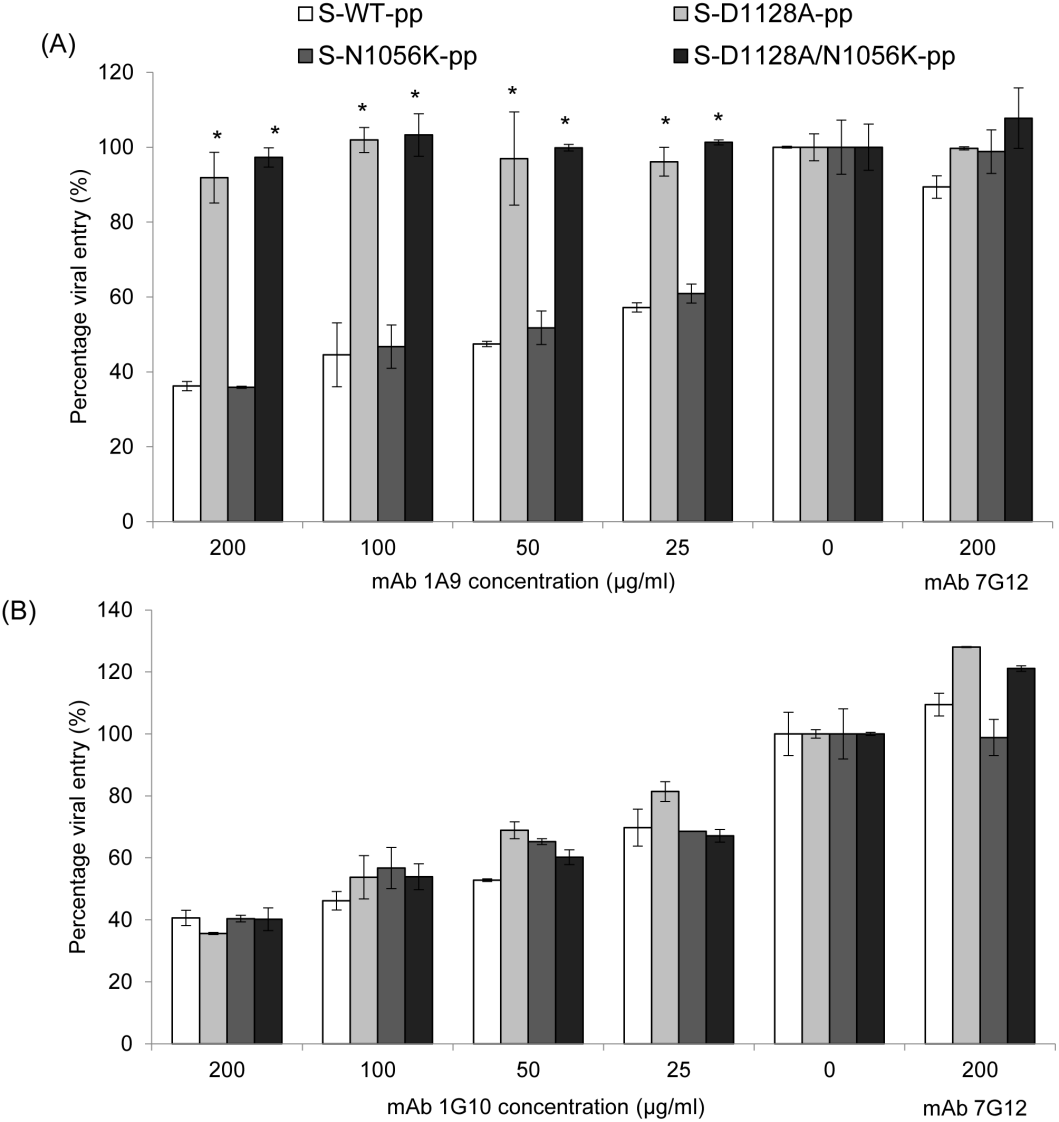
Neutralization of wild-type, mutant D1128A, N1056K and D1128A/N1056K S-pps by mAb 1A9 and mAb 1G10. S-pp containing wild-type (S-WT-pp) or mutant S (S-D1128A-pp, S-N1056K-pp and S-D1128A/N1056K-pp) were pre-incubated with different concentrations of (A) mAb 1A9 or (B) mAb 1G10 at 25, 50, 100 and 200 µg/ml for 1 hour before infecting CHO-ACE2 cells. An anti-S1, non-neutralizing mAb 7G12 was used as control antibody at 200 µg/ml. Cells were harvested 48 hours post-infection and luciferase activities were measured. Viral entry, as indicated by the luciferase activity measured in relative light units (RLU), was expressed as a percentage of the reading obtained in the absence of antibody, which was set at 100%. Data shown represents that obtained from 3 independent experiments. Bars represent SD of the experiment carried out in triplicates. *indicates statistically significant difference of *p*<0. 01 when compared to S-WT-pp.

Both D1128A and N1056K are mutations identified in escape SARS-CoV mutant clones generated against mAb 1A9. To verify if these two mutations contribute to the escape from neutralization by mAb 1A9 in an *in vitro* pseudotyped virus assay, S-pps expressing the wild-type, mutant D1128A, mutant N1056K and mutant D1128A/N1056K S proteins were generated. As seen in Figure S1B in File S1, all S-pps were able to infect and enter CHO-ACE2 cells, with the mutant S-pps showing a slightly lower infectivity compared to wild-type. They were then used to infect CHO-ACE2 cells in the absence or presence of different concentrations (25, 50, 100 and 200 µg/ml) of mAb 1A9 and the neutralization activities of mAb 1A9 against wild-type S-pp, S-D1128A-pp, S-N1056K-pp and S-D1128A/N1056K-pp were compared. MAb 7G12, an anti-S1, non-neutralizing mAb, was used as the control antibody at 200 µg/ml. As shown in Figure 6A and Figure S5A in File S1, at the highest concentration of 200 µg/ml, mAb 1A9 prevented the viral entry of wild-type S-pp and S-N1056K-pp in CHO-ACE2 cells by 36% and 35% respectively, while the entry of S-D1128A-pp was not significantly affected. At lower concentrations of mAb 1A9 (25, 50 and 100 µg/ml), similar results were obtained, suggesting that S-D1128-pp was resistant to mAb 1A9 neutralization. This further indicates that through a reduction in binding to mAb 1A9 (as observed in Western Blot, IP and ELISA), the D1128A mutation in S protein is sufficient to mediate the escape from neutralization by mAb 1A9. In addition, S-pp containing both D1128A and N1056K mutations was investigated for its resistance to mAb 1A9 neutralization. S-D1128A/N1056K-pp was also resistant to mAb 1A9 neutralization at a similar extent as the S-D1128A-pp (Figure 6A and Figure S5A in File S1), indicating no synergistic effects between the D1128A and N1056K mutations in conferring mAb 1A9 resistance to the viral particles. No resistance to neutralization was observed for all S-pp with mAb 1G10, another neutralizing mAb that binds to a different epitope within the S2 subunit at amino acids 1151–1192 (Figure 6B and Figure S5B in File S1).

### Effects of D1128A mutation on the expression of S protein on cell surface and incorporation of S protein incorporation into S-pp

**Figure 7:**
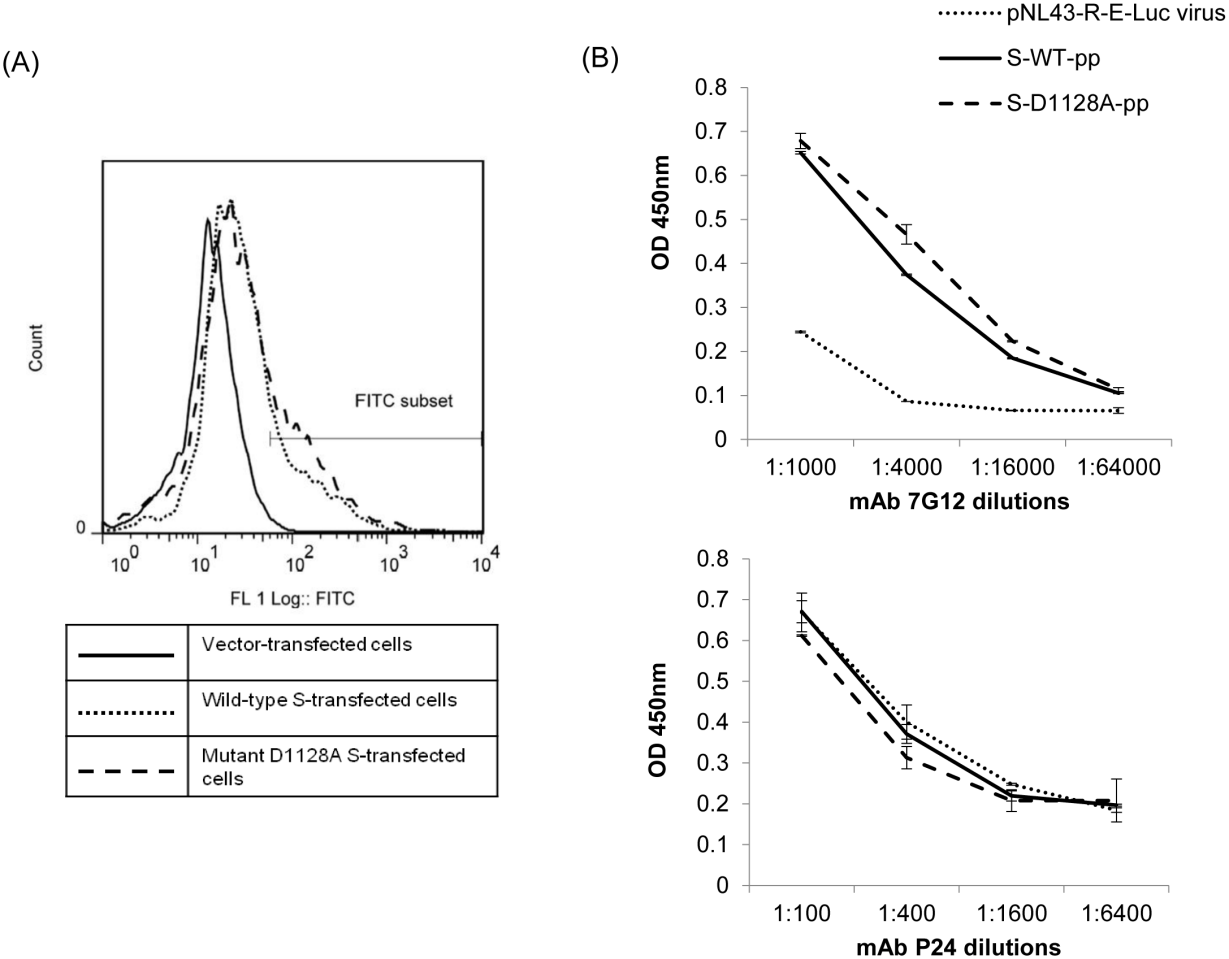
Determination of cell surface expression of wild-type and mutant D1128A S proteins by FACS analysis and determination of wild-type and mutant D1128A S protein incorporation in S-pp by ELISA. (A) FACS analysis was performed with 293 FT cells were transfected with empty vector (full line) or with plasmids expressing full length wild-type S (dotted line) or full length mutant D1128A S (dash line). Cells were harvested 72 hours post-transfection and stained with mouse mAb 7G12, followed by FITC-conjugated goat anti-mouse antibody. Results shown are representative of 3 independent experiments. (B) Pseudoviral particles not expressing S (pNL43-R-E-Luc virus), S-pp expressing wild-type S (S-WT-pp) and mutant D1128A S (S-D1128A-pp) were coated on a 96-well plate at 16 ng/well, as previously quantitated using P24 ELISA, and detected using mAb 7G12 (top) and mAb P24 (bottom) at 4-fold serial dilutions. Optical density (OD) was measured at 450 nm. Bars represent SD of the experiment carried out in triplicates. MAb P24 was used as a control antibody to ensure equal amounts of S-pp were coated onto each well.

Mutations within the coronavirus S protein can have profound effects on the synthesis and maturation process of the S protein, resulting in decreased cell surface expression as well as defects in its incorporation into matured virion particles. To evaluate the possible effects of D1128A mutation on the maturation process of the S protein during its synthesis, FACS analysis was performed to compare the cell surface expression of wild-type (WT) and mutant D1128A S proteins in transfected 293 FT cells. Vector-transfected, wild-type S-transfected and mutant D1128A S-transfected cells were incubated with mAb 7G12 (which binds to the S1 subunit of S) followed by an FITC-conjugated anti-mouse secondary antibody. A positive shift in fluorescence was observed in the wild-type S-expressing cells when compared to the vector-transfected cells (Figure 7A) because of the specific binding of mAb 7G12 to the native form of the S protein expressed on the cell surface. In comparison, the mutant D1128A S-expressing cells showed similar degree of shift in fluorescence as those expressing wild-type S (Figure 7A). Thus, the D1128A mutation did not reduce the surface level expression of the S protein, suggesting that this substitution at residue 1128 did not hamper the synthesis and processing of the S protein.

To determine if the mutant D1128A S protein is incorporated into the S-pps as efficiently as wild-type S, equal amount of wild-type S-pp and S-D1128A-pp was coated onto an ELISA plate and mAb 7G12 was used to compare the amount of S protein in the S-pps. It was observed that there was no significant difference in the expression of wild-type and mutant D1128A S protein in the S-pps (Figure 7B, top). This indicates that the mutation did not cause any change in the efficiency of S protein incorporation into viral particles. When an anti-HIV-1 P24 mAb was used instead of mAb 7G12, the OD450 nm readings obtained were similar, confirming that equal amounts of S-pp viruses were successfully coated on the ELISA plate (Figure 7B, bottom).

## Discussion

Although there has been no reported case of SARS-CoV infection in humans since 2004, the development of anti-SARS-CoV treatments and vaccines remains crucial as the threat of a re-emergence of SARS exists till today. Human and civet SARS-CoVs are believed to have originated from SL-CoVs residing in bats [42]. As coronaviruses are known to be capable of frequent cross-species transmission [12], the continual persistence of SL-CoVs in animal hosts and reservoirs poses a threat to humans should a cross-species transmission occurs. The development of broadly neutralizing mAbs that confer cross-protection not only against human SARS-CoV, but also zoonotic strains of SARS-CoV and SL-CoV, is therefore important. The putative S1 subunit of bats SL-CoVs has a low sequence homology of about 63% to that of SARS-CoV, especially in the RBD, indicating the usage of different host cell receptors and different tissue tropisms [43]. On the other hand, the high sequence homology in the S2 subunit of about 92–96% suggests that the fusion mechanism during viral infection is well-conserved [44]. Broadly neutralizing mAbs usually target conserved epitopes required for highly conserved process, such as the post-attachment fusion process [45]. A majority of SARS-CoV-neutralizing mAbs reported bind and target the S1 protein at the RBD region [46]. Nonetheless, neutralizing mAbs that bind to the S2 subunit have been reported. The epitopes of these mAbs are found to be located at the S2 subunit upstream of HR1 (residues 787–809, 791–805) [47], [48], within the loop region in between HR1 and HR2 (residues 1023–1189) [31], [40] and within the HR2 domain (residues 1151–1192) [31]. It has also been shown that anti-S2 mAbs that bind to the highly conserved HR1, HR2 and ectodomain of the SARS-CoV S protein were able to neutralize a wider range of clinical isolates, including human and zoonotic strains of SARS-CoVs [28], [30]. In this current study, mAb 1A9, an anti-S2 mAb that binds to the S2 subunit at the highly conserved loop region at residues 1111–1130, was demonstrated to be able to cross-neutralize pseudotyped S-pp viruses of the human SARS-CoV, civet SARS-CoV and bat SL-CoV strains. This is consistent with the sequence conservation of the mAb 1A9 binding epitope in S. In addition, sequence alignment (not shown) revealed that the mAb 1A9 binding site is also conserved in other bat CoVs such as the Bulgarian SARS-related CoV strain [49], indicating the potential cross-protective effect of mAb 1A9 against not only bat SL-CoV from China, but also from other parts of the world such as Europe.

Several cross-neutralizing mAbs against human and civet SARS-CoV strains targeting the RBD have been described, with IC50 values ranging from 0.01 to 0.5 µg/ml [38], . As observed in this study, the IC50 value of mAb 1A9 is between 25–50 µg/ml, which is higher than that of RBD-targeting mAbs, indicating the lower potency of mAb 1A9. Higher IC50 values and lower potency have also been observed in several other anti-S2 mAbs [40], [52]. This can possibly be attributed to the inaccessibility of the S2 subunit, which constitutes the stalk region of S, as compared to the S1 subunit that is exposed on the viral surface [53]. However, SARS-CoV is capable of attaining mutations in the RBD region without affecting viral infectivity [54], leading to escape in mAb neutralization, while mutations within the highly conserved S2 subunit region are more likely to be detrimental to the mutant virus. Therefore, the characterization of anti-S2 mAbs remains important in the development of antibodies as anti-viral therapies against SARS-CoV.

Through the generation of mAb 1A9 escape virus, it was found that the escape mutation D1128A in the S protein resulted in diminished binding to mAb 1A9 and S pseudotyped viral particles containing the D1128A mutation could not be neutralized by mAb 1A9. In addition, the substitution of D1128 by either N (having same side-chain as D) or E (having same charge as D) also reduced the interaction with mAb 1A9 to similar extent as the A substitution (Figure S6 in File S1). Thus, it appears that the D residue at position 1128 in the S protein plays an essential role in the interaction with mAb 1A9. Another mutation, N1056K, also identified in mAb 1A9 escape virus, was found to have no effects on mAb 1A9 binding and neutralization, indicating that this is most likely a random mutation that arose during the generation of escape mutants. No significant synergistic effect was observed between the D1128A and N1056K mutations in decreasing mAb 1A9 binding and conferring resistance to mAb 1A9 neutralization. As there is no information on the possible functional role of the mAb 1A9 binding epitope or the residue D1128, the cell surface expression of mutant S-D1128A and its incorporation into viral-like particles was compared to that of wild-type S. The results showed that S-D1128A is similar to wild-type S in these aspects, suggesting that viral entry property of the escape virus has not changed. In summary, we characterized mAb 1A9, a SARS-CoV neutralizing mAb that binds to a novel epitope located within the loop region located in between HR1 and HR2, at a position directly upstream of HR2 at residues 1111–1130 of the S protein that has not been previously identified and characterized. The aspartic acid at residue 1128 is crucial for the interaction of S protein with mAb 1A9 and a substitution to alanine in the escape virus is sufficient to abolish neutralization by mAb 1A9 but has little effect on the viral entry property. Consistently, while a detailed study on the fitness of the escape virus has not been performed, it was observed that the virus titre of the escape virus after 3 passages in presence of mAb 1A9 reached similar level as the wild-type virus (see Figure 3). However, we cannot rule out the possibility that the mutation may affect viral virulence and further work is needed to address this. The loop region in between HR1 and HR2 in the S2 subunit is believed to be a region required for viral-cell membrane fusion, as peptides analogous to the loop region were found to inhibit SARS-CoV infection [55]. Although D1128 residue has not been shown to be directly involved in membrane fusion, it is probable that the binding of mAb 1A9 to the D1128 residue in the loop region causes steric hindrance that prevents the association of HR1 and HR2 to form the six-helical fusion bundle core. Hence, structural analysis of the interaction between mAb 1A9 and S is actively being pursued in order to define the inhibition mechanism of mAb 1A9.

## Supporting Information

File S1 **Supporting information Figures S1–S6.** Figure S1. Infectivity of pseudotyped viruses expressing S protein (S-pps). (A) S-pp expressing S protein of humans SARS-CoV HKU39849, civet SARS-CoV SZ3, bat SL-CoV Rp3 and Rf1 and (B) S-pp containing wild-type or mutant D1128A, N1056K or D1128A/N1056K S were generated and used to infect CHO-ACE2 cells at equal amount (as quantitated using P24 ELISA). Cells were harvested 48 hours post-infection and luciferase readings were measured. pNL43-R-E-Luc virus, which do not express S protein, was used as negative control. Error bars represent SD of experiment carried out in triplicates. Figure S2. Neutralization of human SARS-CoV HKU39849, civet SARS-CoV SZ3, bat SL-CoV Rp3 and Rf1 S-pps by mAb 1A9 (data presented using absolute luciferase readings). S-pps containing S of human SARS-CoV HKU39849, civet SARS-CoV SZ3, bat SL-CoV Rp3 or Rf1, were pre-incubated with different concentrations of mAb 1A9 at 100, 150 and 200 µg/ml for 1 hour before infecting CHO-ACE2 cells. Cells were harvested 48 hours post-infection and luciferase activities were measured. Data shown represents that obtained from 3 independent experiments. Bars represent SD of the experiment carried out in triplicates. Figure S3. Binding of mAb 1A9 to wild-type, mutant D1128A and N1056K GST-S(1030-1188) fragments by ELISA. In ELISA, GST, GST-S(1030-1188) wild-type, GST-S(1030-1188)-D1128A and GST-S(1030-1188)-N1056K proteins were coated on a 96-well plate at 100 ng/well and detected using (C) mAb 1A9 and (D) mAb GST at 4-fold serial dilutions. Optical density (OD) was measured at 450 nm. Bars represent SD of the experiment carried out in triplicates. *indicates statistically significant difference (*p*<0.01) when compared to wild-type. MAb GST was used as a control antibody to ensure that equal amounts of GST-tagged proteins were coated onto each well. A significant reduction of mAb 1A9 binding was observed for S fragment containing the D1128A mutation (*p*<0.01). Figure S4. Expression and purification of wild-type and mutant GST-S(1030-1188) fragments. Purified GST, GST-S(1030-1188) wild-type, GST-S(1030-1188)-D1128A and GST-S(1030-1188)-N1056K fragments (lanes 1–4) were separated on a 12% gel by SDS-PAGE and stained using Commassie Blue. Molecular weight markers in kDa are indicated on the left. Expected size of each GST-S(1030-1188) fragment is around 43 kDa as indicated by the arrow. Figure S5. Neutralization of wild-type and mutant D1128A, N1056K and D1128A/N1056K S-pps by mAb 1A9 and mAb 1G10 (data presented using absolute luciferase readings). S-pp containing wild-type (S-WT-pp) or mutant S (S-D1128A-pp, S-N1056K-pp and S-D1128A/N1056K-pp) were pre-incubated with different concentrations of (A) mAb 1A9 and (B) mAb 1G10 at 25, 50, 100 and 200 µg/ml for 1 hour before infecting CHO-ACE2 cells. An anti-S1, non-neutralizing mAb 7G12 was used as control antibody at 200 µg/ml. Cells were harvested 48 hours post-infection and luciferase activities were measured. Data shown represents that obtained from 3 independent experiments. Bars represent SD of the experiment carried out in triplicates. *indicates statistically significant difference of *p*<0. 01 when compared to S-WT-pp. Figure S6. Binding of mAb 1A9 to wild-type, mutant D1128A, D1128E, D1128N and N1056K S proteins. 293 FT cells were transfected with no plasmid (mock) or with plasmids expressing full length wild-type S (S-WT) or full length mutant S (S-D1128A, S-D1128E, S-D1128N and S-N1056K). Western Blot analysis was performed on the cell lysates using mAb 1A9 to determine its binding to the various S proteins. MAb 7G12, which binds to the S1 of the S protein, was used as a control antibody to detect protein expression.
(ZIP)
