# Supplementary figures and images for: Substitution at Aspartic Acid 1128 in the SARS Coronavirus Spike Glycoprotein Mediates Escape from a S2 Domain-Targeting Neutralizing Monoclonal Antibody
Source: PLoS One. 2014 Jul 14;9(7):e102415. doi: 10.1371/journal.pone.0102415 (PMC4097068; doi:10.1371/journal.pone.0102415)

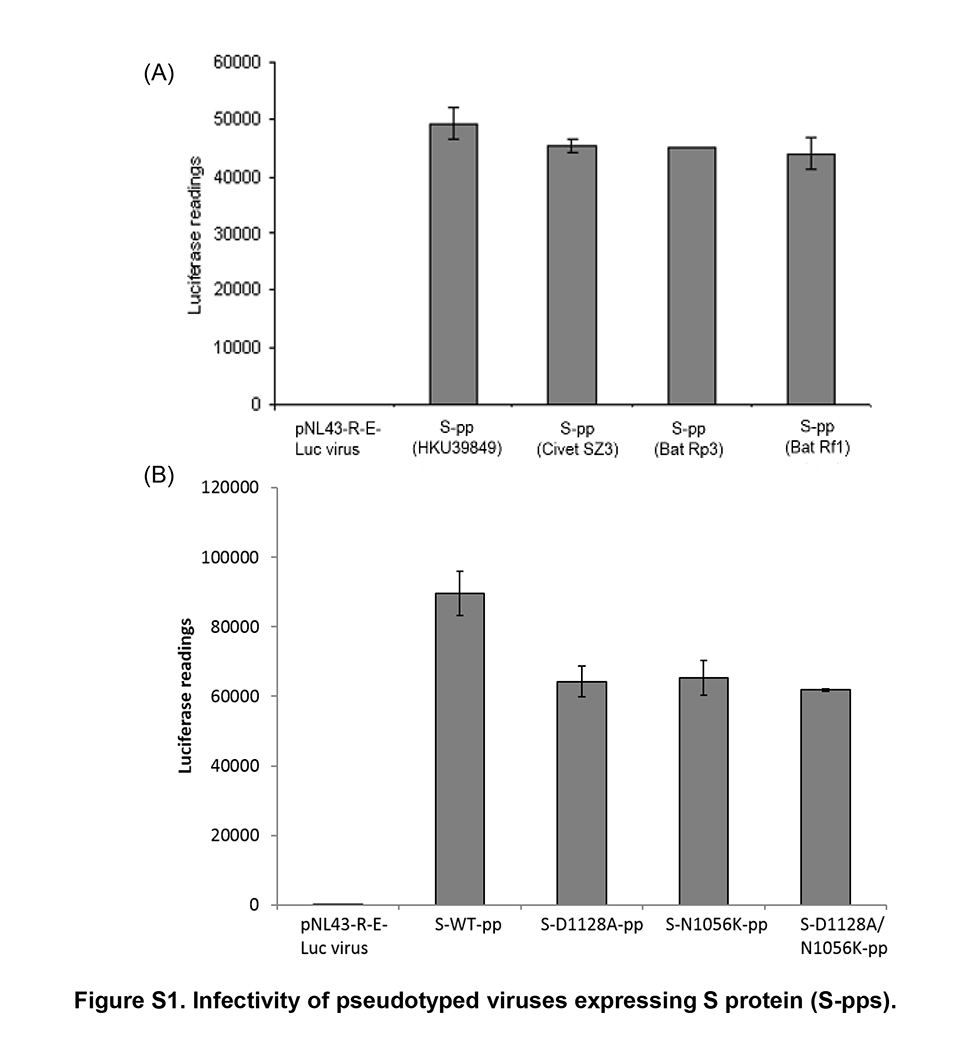

Supplement: File S1 — Supporting information Figures S1–S6. Figure S1. Infectivity of pseudotyped viruses expressing S protein (S-pps). (A) S-pp expressing S protein of humans SARS-CoV HKU39849, civet SARS-CoV SZ3, bat SL-CoV Rp3 and Rf1 and (B) S-pp containing wild-type or mutant D1128A, N1056K or D1128A/N1056K S were generated and used to infect CHO-ACE2 cells at equal amount (as quantitated using P24 ELISA). Cells were harvested 48 hours post-infection and luciferase readings were measured. pNL43-R-E-Luc virus, which do not express S protein, was used as negative control. Error bars represent SD of experiment carried out in triplicates. Figure S2. Neutralization of human SARS-CoV HKU39849, civet SARS-CoV SZ3, bat SL-CoV Rp3 and Rf1 S-pps by mAb 1A9 (data presented using absolute luciferase readings). S-pps containing S of human SARS-CoV HKU39849, civet SARS-CoV SZ3, bat SL-CoV Rp3 or Rf1, were pre-incubated with different concentrations of mAb 1A9 at 100, 150 and 200 µg/ml for 1 hour before infecting CHO-ACE2 cells. Cells were harvested 48 hours post-infection and luciferase activities were measured. Data shown represents that obtained from 3 independent experiments. Bars represent SD of the experiment carried out in triplicates. Figure S3. Binding of mAb 1A9 to wild-type, mutant D1128A and N1056K GST-S(1030-1188) fragments by ELISA. In ELISA, GST, GST-S(1030-1188) wild-type, GST-S(1030-1188)-D1128A and GST-S(1030-1188)-N1056K proteins were coated on a 96-well plate at 100 ng/well and detected using (C) mAb 1A9 and (D) mAb GST at 4-fold serial dilutions. Optical density (OD) was measured at 450 nm. Bars represent SD of the experiment carried out in triplicates. *indicates statistically significant difference (p<0.01) when compared to wild-type. MAb GST was used as a control antibody to ensure that equal amounts of GST-tagged proteins were coated onto each well. A significant reduction of mAb 1A9 binding was observed for S fragment containing the D1128A mutation (p<0.01). Figure S4. Ex [file pone.0102415.s001.zip › Updated S figures/File S1.tif]

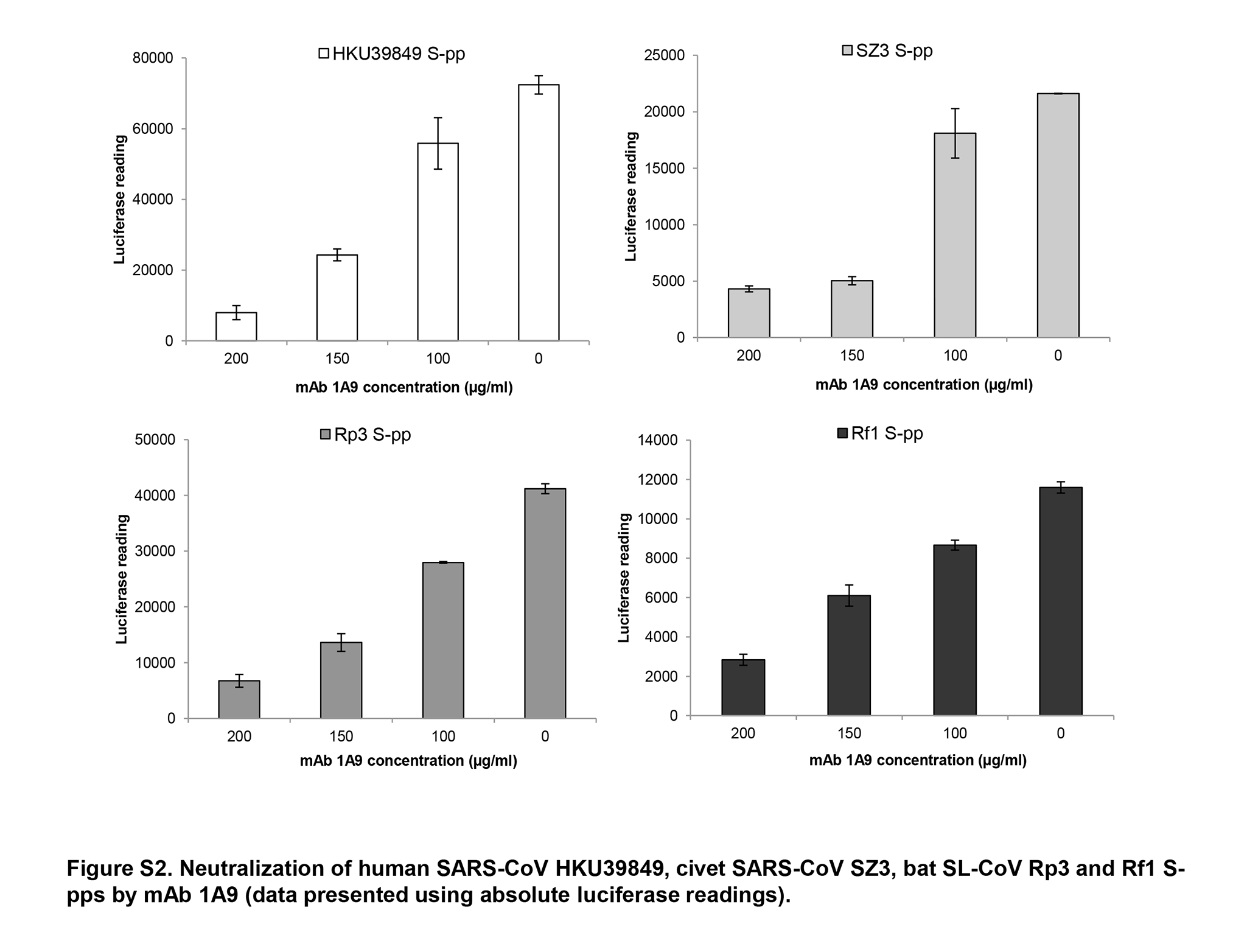

Supplement: File S1 — Supporting information Figures S1–S6. Figure S1. Infectivity of pseudotyped viruses expressing S protein (S-pps). (A) S-pp expressing S protein of humans SARS-CoV HKU39849, civet SARS-CoV SZ3, bat SL-CoV Rp3 and Rf1 and (B) S-pp containing wild-type or mutant D1128A, N1056K or D1128A/N1056K S were generated and used to infect CHO-ACE2 cells at equal amount (as quantitated using P24 ELISA). Cells were harvested 48 hours post-infection and luciferase readings were measured. pNL43-R-E-Luc virus, which do not express S protein, was used as negative control. Error bars represent SD of experiment carried out in triplicates. Figure S2. Neutralization of human SARS-CoV HKU39849, civet SARS-CoV SZ3, bat SL-CoV Rp3 and Rf1 S-pps by mAb 1A9 (data presented using absolute luciferase readings). S-pps containing S of human SARS-CoV HKU39849, civet SARS-CoV SZ3, bat SL-CoV Rp3 or Rf1, were pre-incubated with different concentrations of mAb 1A9 at 100, 150 and 200 µg/ml for 1 hour before infecting CHO-ACE2 cells. Cells were harvested 48 hours post-infection and luciferase activities were measured. Data shown represents that obtained from 3 independent experiments. Bars represent SD of the experiment carried out in triplicates. Figure S3. Binding of mAb 1A9 to wild-type, mutant D1128A and N1056K GST-S(1030-1188) fragments by ELISA. In ELISA, GST, GST-S(1030-1188) wild-type, GST-S(1030-1188)-D1128A and GST-S(1030-1188)-N1056K proteins were coated on a 96-well plate at 100 ng/well and detected using (C) mAb 1A9 and (D) mAb GST at 4-fold serial dilutions. Optical density (OD) was measured at 450 nm. Bars represent SD of the experiment carried out in triplicates. *indicates statistically significant difference (p<0.01) when compared to wild-type. MAb GST was used as a control antibody to ensure that equal amounts of GST-tagged proteins were coated onto each well. A significant reduction of mAb 1A9 binding was observed for S fragment containing the D1128A mutation (p<0.01). Figure S4. Ex [file pone.0102415.s001.zip › Updated S figures/File S2.tif]

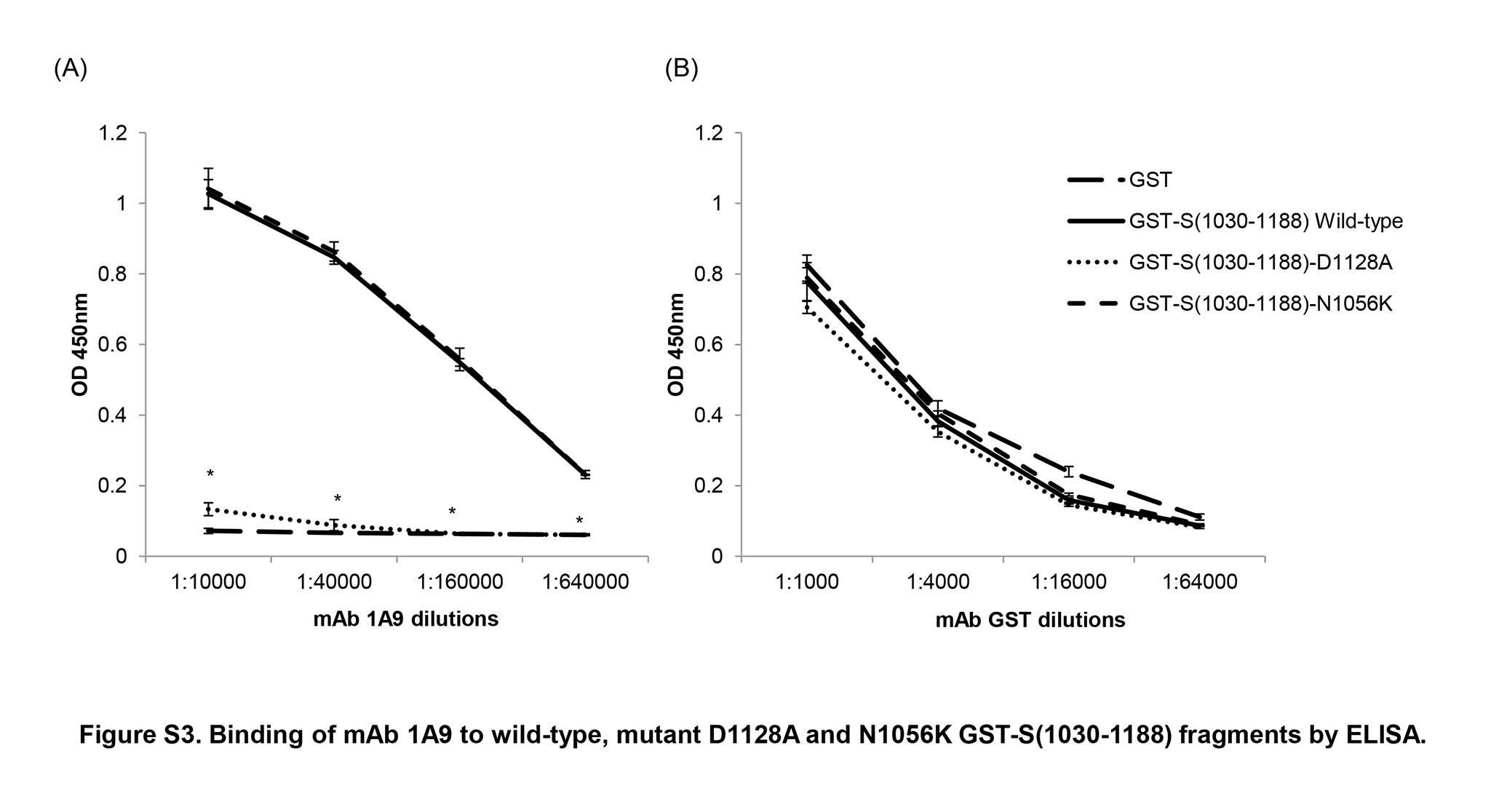

Supplement: File S1 — Supporting information Figures S1–S6. Figure S1. Infectivity of pseudotyped viruses expressing S protein (S-pps). (A) S-pp expressing S protein of humans SARS-CoV HKU39849, civet SARS-CoV SZ3, bat SL-CoV Rp3 and Rf1 and (B) S-pp containing wild-type or mutant D1128A, N1056K or D1128A/N1056K S were generated and used to infect CHO-ACE2 cells at equal amount (as quantitated using P24 ELISA). Cells were harvested 48 hours post-infection and luciferase readings were measured. pNL43-R-E-Luc virus, which do not express S protein, was used as negative control. Error bars represent SD of experiment carried out in triplicates. Figure S2. Neutralization of human SARS-CoV HKU39849, civet SARS-CoV SZ3, bat SL-CoV Rp3 and Rf1 S-pps by mAb 1A9 (data presented using absolute luciferase readings). S-pps containing S of human SARS-CoV HKU39849, civet SARS-CoV SZ3, bat SL-CoV Rp3 or Rf1, were pre-incubated with different concentrations of mAb 1A9 at 100, 150 and 200 µg/ml for 1 hour before infecting CHO-ACE2 cells. Cells were harvested 48 hours post-infection and luciferase activities were measured. Data shown represents that obtained from 3 independent experiments. Bars represent SD of the experiment carried out in triplicates. Figure S3. Binding of mAb 1A9 to wild-type, mutant D1128A and N1056K GST-S(1030-1188) fragments by ELISA. In ELISA, GST, GST-S(1030-1188) wild-type, GST-S(1030-1188)-D1128A and GST-S(1030-1188)-N1056K proteins were coated on a 96-well plate at 100 ng/well and detected using (C) mAb 1A9 and (D) mAb GST at 4-fold serial dilutions. Optical density (OD) was measured at 450 nm. Bars represent SD of the experiment carried out in triplicates. *indicates statistically significant difference (p<0.01) when compared to wild-type. MAb GST was used as a control antibody to ensure that equal amounts of GST-tagged proteins were coated onto each well. A significant reduction of mAb 1A9 binding was observed for S fragment containing the D1128A mutation (p<0.01). Figure S4. Ex [file pone.0102415.s001.zip › Updated S figures/File S3.tif]

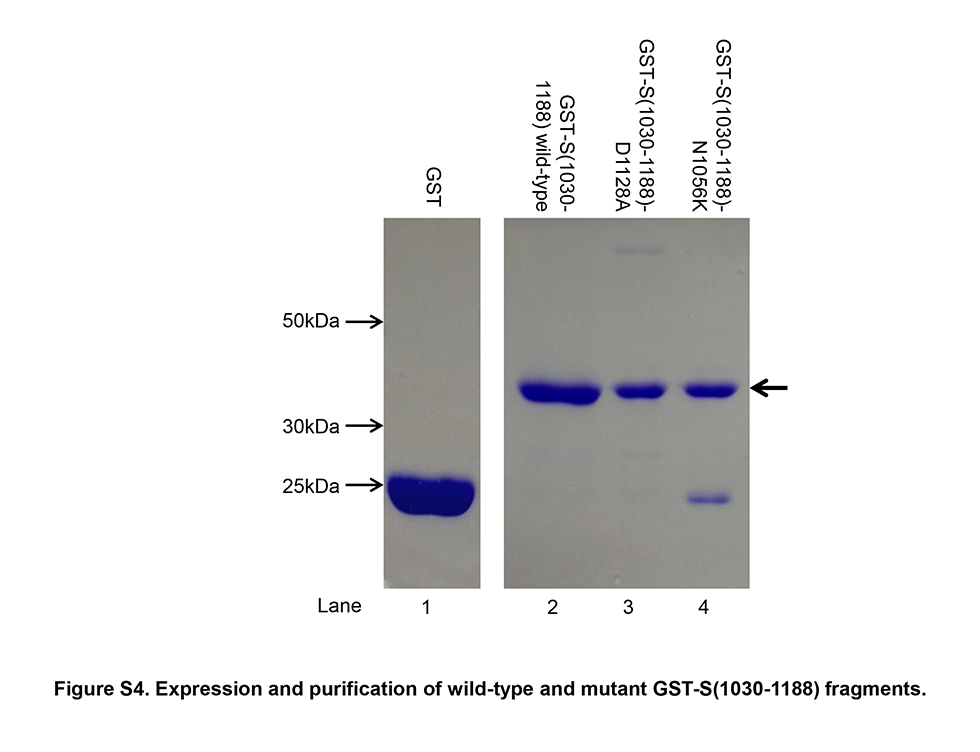

Supplement: File S1 — Supporting information Figures S1–S6. Figure S1. Infectivity of pseudotyped viruses expressing S protein (S-pps). (A) S-pp expressing S protein of humans SARS-CoV HKU39849, civet SARS-CoV SZ3, bat SL-CoV Rp3 and Rf1 and (B) S-pp containing wild-type or mutant D1128A, N1056K or D1128A/N1056K S were generated and used to infect CHO-ACE2 cells at equal amount (as quantitated using P24 ELISA). Cells were harvested 48 hours post-infection and luciferase readings were measured. pNL43-R-E-Luc virus, which do not express S protein, was used as negative control. Error bars represent SD of experiment carried out in triplicates. Figure S2. Neutralization of human SARS-CoV HKU39849, civet SARS-CoV SZ3, bat SL-CoV Rp3 and Rf1 S-pps by mAb 1A9 (data presented using absolute luciferase readings). S-pps containing S of human SARS-CoV HKU39849, civet SARS-CoV SZ3, bat SL-CoV Rp3 or Rf1, were pre-incubated with different concentrations of mAb 1A9 at 100, 150 and 200 µg/ml for 1 hour before infecting CHO-ACE2 cells. Cells were harvested 48 hours post-infection and luciferase activities were measured. Data shown represents that obtained from 3 independent experiments. Bars represent SD of the experiment carried out in triplicates. Figure S3. Binding of mAb 1A9 to wild-type, mutant D1128A and N1056K GST-S(1030-1188) fragments by ELISA. In ELISA, GST, GST-S(1030-1188) wild-type, GST-S(1030-1188)-D1128A and GST-S(1030-1188)-N1056K proteins were coated on a 96-well plate at 100 ng/well and detected using (C) mAb 1A9 and (D) mAb GST at 4-fold serial dilutions. Optical density (OD) was measured at 450 nm. Bars represent SD of the experiment carried out in triplicates. *indicates statistically significant difference (p<0.01) when compared to wild-type. MAb GST was used as a control antibody to ensure that equal amounts of GST-tagged proteins were coated onto each well. A significant reduction of mAb 1A9 binding was observed for S fragment containing the D1128A mutation (p<0.01). Figure S4. Ex [file pone.0102415.s001.zip › Updated S figures/File S4.tif]

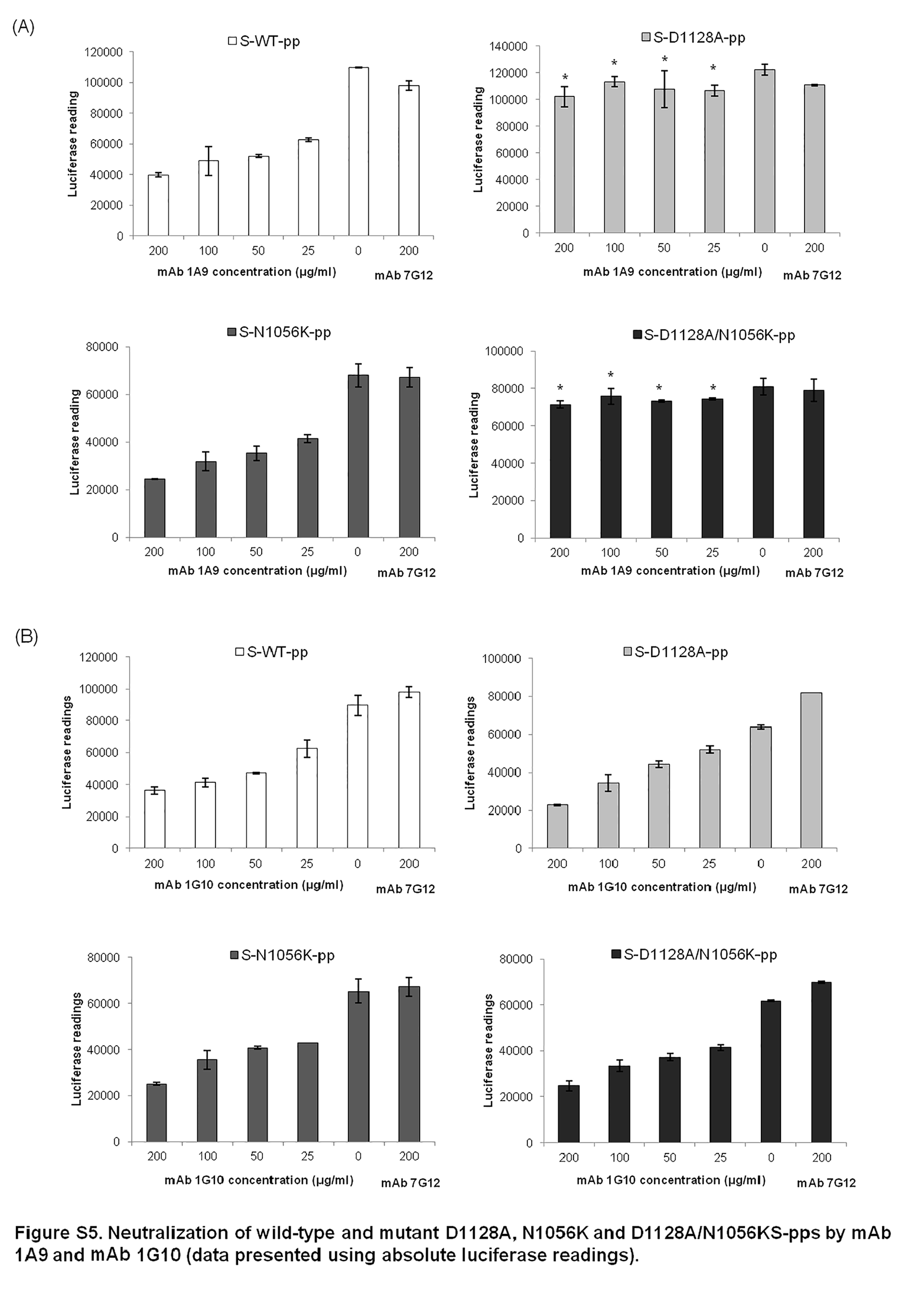

Supplement: File S1 — Supporting information Figures S1–S6. Figure S1. Infectivity of pseudotyped viruses expressing S protein (S-pps). (A) S-pp expressing S protein of humans SARS-CoV HKU39849, civet SARS-CoV SZ3, bat SL-CoV Rp3 and Rf1 and (B) S-pp containing wild-type or mutant D1128A, N1056K or D1128A/N1056K S were generated and used to infect CHO-ACE2 cells at equal amount (as quantitated using P24 ELISA). Cells were harvested 48 hours post-infection and luciferase readings were measured. pNL43-R-E-Luc virus, which do not express S protein, was used as negative control. Error bars represent SD of experiment carried out in triplicates. Figure S2. Neutralization of human SARS-CoV HKU39849, civet SARS-CoV SZ3, bat SL-CoV Rp3 and Rf1 S-pps by mAb 1A9 (data presented using absolute luciferase readings). S-pps containing S of human SARS-CoV HKU39849, civet SARS-CoV SZ3, bat SL-CoV Rp3 or Rf1, were pre-incubated with different concentrations of mAb 1A9 at 100, 150 and 200 µg/ml for 1 hour before infecting CHO-ACE2 cells. Cells were harvested 48 hours post-infection and luciferase activities were measured. Data shown represents that obtained from 3 independent experiments. Bars represent SD of the experiment carried out in triplicates. Figure S3. Binding of mAb 1A9 to wild-type, mutant D1128A and N1056K GST-S(1030-1188) fragments by ELISA. In ELISA, GST, GST-S(1030-1188) wild-type, GST-S(1030-1188)-D1128A and GST-S(1030-1188)-N1056K proteins were coated on a 96-well plate at 100 ng/well and detected using (C) mAb 1A9 and (D) mAb GST at 4-fold serial dilutions. Optical density (OD) was measured at 450 nm. Bars represent SD of the experiment carried out in triplicates. *indicates statistically significant difference (p<0.01) when compared to wild-type. MAb GST was used as a control antibody to ensure that equal amounts of GST-tagged proteins were coated onto each well. A significant reduction of mAb 1A9 binding was observed for S fragment containing the D1128A mutation (p<0.01). Figure S4. Ex [file pone.0102415.s001.zip › Updated S figures/File S5.tif]

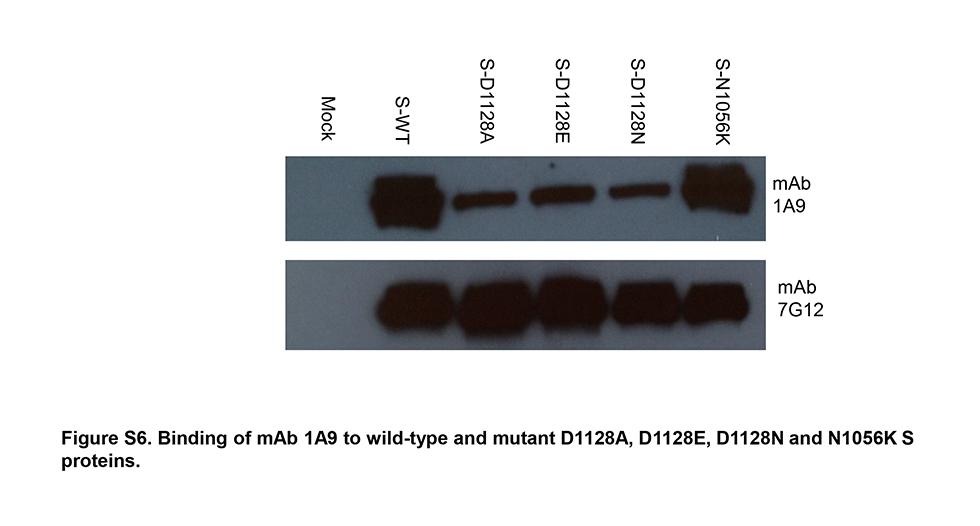

Supplement: File S1 — Supporting information Figures S1–S6. Figure S1. Infectivity of pseudotyped viruses expressing S protein (S-pps). (A) S-pp expressing S protein of humans SARS-CoV HKU39849, civet SARS-CoV SZ3, bat SL-CoV Rp3 and Rf1 and (B) S-pp containing wild-type or mutant D1128A, N1056K or D1128A/N1056K S were generated and used to infect CHO-ACE2 cells at equal amount (as quantitated using P24 ELISA). Cells were harvested 48 hours post-infection and luciferase readings were measured. pNL43-R-E-Luc virus, which do not express S protein, was used as negative control. Error bars represent SD of experiment carried out in triplicates. Figure S2. Neutralization of human SARS-CoV HKU39849, civet SARS-CoV SZ3, bat SL-CoV Rp3 and Rf1 S-pps by mAb 1A9 (data presented using absolute luciferase readings). S-pps containing S of human SARS-CoV HKU39849, civet SARS-CoV SZ3, bat SL-CoV Rp3 or Rf1, were pre-incubated with different concentrations of mAb 1A9 at 100, 150 and 200 µg/ml for 1 hour before infecting CHO-ACE2 cells. Cells were harvested 48 hours post-infection and luciferase activities were measured. Data shown represents that obtained from 3 independent experiments. Bars represent SD of the experiment carried out in triplicates. Figure S3. Binding of mAb 1A9 to wild-type, mutant D1128A and N1056K GST-S(1030-1188) fragments by ELISA. In ELISA, GST, GST-S(1030-1188) wild-type, GST-S(1030-1188)-D1128A and GST-S(1030-1188)-N1056K proteins were coated on a 96-well plate at 100 ng/well and detected using (C) mAb 1A9 and (D) mAb GST at 4-fold serial dilutions. Optical density (OD) was measured at 450 nm. Bars represent SD of the experiment carried out in triplicates. *indicates statistically significant difference (p<0.01) when compared to wild-type. MAb GST was used as a control antibody to ensure that equal amounts of GST-tagged proteins were coated onto each well. A significant reduction of mAb 1A9 binding was observed for S fragment containing the D1128A mutation (p<0.01). Figure S4. Ex [file pone.0102415.s001.zip › Updated S figures/File S6.tif]
